# Changes in Blood Factors and Ultrasound Findings in Mild Cognitive Impairment and Dementia

**DOI:** 10.3389/fnagi.2017.00427

**Published:** 2017-12-21

**Authors:** Kyoungjoo Cho, Jihye Kim, Gyung W. Kim

**Affiliations:** Department of Neurology, College of Medicine, Yonsei University, Seoul, South Korea

**Keywords:** dementia, vascular disease, mild cognitive impairment, blood factor analysis, atherosclerosis

## Abstract

The present study aimed to assess the changes in blood factors and ultrasound measures of atherosclerosis burden patient with mild cognitive impairment (MCI) and dementia. Peripheral blood samples and ultrasonography findings were obtained for 53 enrolled participants. Flow cytometry was used to evaluate levels of activated platelets and platelet-leukocyte aggregates (PLAs). The number of platelets expressing p-selectin was correlated with intima media thickness (IMT) and plaque number in both the MCI and dementia groups. The number of platelets expressing p-selectin glycoprotein ligand (PSGL) was strongly correlated with IMT in patients with MCI, whereas the number of platelets expressing PGSL was correlated with plaque number rather than IMT in patients with dementia. PLAs was associated with both IMT and plaque number in patients with MCI but not in those with dementia. Our findings demonstrate that alterations in IMT and plaque number are associated with an increased risk of cognitive decline as well as conversion from MCI to dementia and that blood factor analysis may aid to detect the severity of cognitive decline.

## Introduction

Aging has been regarded as a major risk factor for neurodegenerative disease (Coutu et al., 2017). Previous studies have demonstrated a strong association between aging and vascular diseases (Jeerakathil et al., 2004; Gottesman et al., 2010; Pantoni, 2010). The research has indicated that factors associated with aging and vascular dysfunction exhibit a cross-sectional relationship with mental status as determined based on Mini-Mental State Examination (MMSE) score (Atiya et al., 2003). Recent studies have reported that carotid artery atherosclerosis is associated with a subsequent risk of new or recurrent cerebrovascular diseases, such as stroke, post-stroke vascular dementia and mild cognitive impairment (MCI; Knopman et al., 2016; Dearborn et al., 2017; Meyer et al., 2017). Furthermore, chronic hypoperfusion caused by carotid stenosis has been reported to play a role in cognitive decline (Ruitenberg et al., 2005; Yurkovetsky et al., 2006).

Dementia represents a major public health concern (Moon et al., 2015), as accumulating evidence has demonstrated that the incidence and prevalence of dementia increase rapidly with advancing age (Silvestrini et al., 2009; Petersen et al., 2014; Moon et al., 2015). Although it has been difficult to investigate changes in the incidence and prevalence of dementia due to variations in diagnostic criteria and methods, a recent epidemiological study indicated that dementia prevalence and incidence have decreased in some countries (Wu et al., 2017). Moreover, the number of patients with dementia remains stable in the aging population of these countries (Wu et al., 2017). Some evidence has suggested that vascular risk factors are associated with the onset and progression of Alzheimer’s disease (AD; Luchsinger et al., 2005; Deschaintre et al., 2009). In addition, increased cerebrovascular risk has been associated with more severe dementia and MCI incidence (Luchsinger et al., 2005; Mioshi et al., 2006; Deschaintre et al., 2009; Li et al., 2011). Considering the role of vascular blood factors in patients with MCI, such factors may also influence the progression of cognitive decline (Iadecola and Gorelick, 2003). However, there are currently no markers for the prediction of prognosis or the risk of conversion from MCI to dementia. Therefore, it is necessary to develop noninvasive diagnostic methods for the assessment of vascular status (de la Torre, 2010).

Recent clinical investigations have focused on the relationship between levels of circulating adhesion factors in peripheral blood and cerebrovascular diseases (Vermeer et al., 2003; Dearborn et al., 2017). Platelets and leukocytes play a major role in atherothrombosis, aggregates of which result in the formation of atherosclerotic plaque (Folsom et al., 2009; Lievens et al., 2010; Dopheide et al., 2016; Gerdes et al., 2016). Although other factors associated with vascular disease can influence cognitive state, few studies have utilized flow cytometry to investigate platelet and leukocyte markers in older adults with cognitive decline. Research has demonstrated a correlation between circulating adhesion molecules in patients with atherosclerosis and atherosclerosis factors such as intima-media thickness (IMT) and the number of plaques, which may aid in determining the presence and/or extent of cognitive decline (Moon et al., 2015). In order to determine the potential usefulness of this correlation for determining diagnoses/prognoses, blood factor analysis is required. Based on the pathophysiological mechanism underlying dementia, most relevant studies have aimed to identify molecular markers based on drug responses (Mioshi et al., 2006; Jellinger and Attems, 2007; Steinhubl et al., 2007; Coley et al., 2008). As such, little is known regarding the potential role of circulating adhesion molecules in patients with vascular diseases during the early and later stages of cognitive dysfunction.

The present study aimed to assess the relationship between changes in blood factors and ultrasound findings in patients with MCI and dementia exhibiting signs of atherosclerosis, and to suggest the possibility of the most appropriate treatment strategy for patients with MCI or multiple diagnoses.

## Materials and Methods

### Participants

The present study enrolled 53 participants who had visited the neurology outpatient clinic of Severance Hospital between August 2016 and February 2017. Participants with an infection such as aspiration pneumonia were excluded to avoid parallel infection, which may also activate platelets or leukocytes. The control group consisted of nine age- and sex-matched individuals with no clinical signs of cerebrovascular or cardiovascular disease such as stenosis or atherosclerosis. Participants of the patient group were categorized into three subgroups: vessel damage, MCI and dementia groups. The “vessel damage” group represents participants who contain one or more cardiovascular and cerebrovascular diseases containing atherosclerosis, stenosis, or stent implanted. “MCI” group is basically classified with based on the Petersen’s criteria (Petersen, 2004). In our case, we only considered the amnestic MCI for more homogeneous sample. The basal score of participants are 25–27 on the MMSE adjusted according to age and education as assessed for the Korean population. Clinical Dementia Rating Scale (CDR) values 0.5 and 1.0 were classified into the MCI group. We also used additional cognitive evaluation battery the Korean version of Addenbrooke’s Cognitive Examination-Revised (K-ACE; Mioshi et al., 2006). The type of “dementia” group was included AD only which is diagnosed by department of neurology in Severance hospital using neuroimaging and cognitive evaluation (MMSE and CDR) containing Seoul Neuropsychological Screening Battery (SNSB) widely used in South Korea.

No differences in atherosclerotic risk profile (e.g., hypertension, hyperlipidemia and diabetes) were observed. Fasting levels of total and high-density lipoprotein (HDL) cholesterol, triglycerides and low-density lipoprotein (LDL) cholesterol were also measured in each participant. The characteristics of the participants are shown in Table 1. All participants underwent cognitive assessment consisting of the CDR scale and MMSE. Patients with MMSE scores from 25 to 27 and CDR values 0.5 and 1.0 were classified into the MCI group. Patients with dementia were diagnosed with dementia and they were classified into the dementia group. All participants provided written informed consent, and the study protocol was approved by the Institutional Review Board of the Severance Hospital (4-2016-0531). All participants gave written informed consent in accordance with the Declaration of Helsinki.

**Table 1:** Patients characteristics.

			Cognitive decline	
	Control participants (*n* = 9)	Vessel damage stenosis patients (*n* = 29)	MCI (*n* = 11)	Dementia (*n* = 14)	*p*-value
Age	65.3 ± 8.1	68.1 ± 12.1	66.3 ± 12.2	70.9 ± 9.7	0.632
Gender					0.188^§^
Male	11.1 (1)	58.6 (17)	54.5 (6)	28.6 (4)	
Female	88.9 (8)	41.4 (12)	45.5 (5)	71.4 (10)	
Hypertension	22.2 (2)	31.0 (9)	45.5 (5)	50.0 (7)	0.821^§^
Diabetes	9.0 (1)	17.2 (5)	27.3 (3)	14.3 (2)	0.420^§^
Hyperlipidemia	22.2 (2)	24.1 (7)	27.3 (3)	50.0 (7)	0.250^§^
Total cholesterol (mg/dL)	168.7 ± 42.9	138.6 ± 43.6	138.9 ± 41.4	152.9 ± 43.0	0.400
LDL cholesterol (mg/dL)	76.9 ± 18.2	69.2 ± 32.4	61.8 ± 38.3	76.9 ± 33.7	0.742
HDL cholesterol (mg/dL)	67.0 ± 40.5	50.3 ± 13.1	54.0 ± 14.9	55.1 ± 17.7	0.344
MMSE score	29 ± 0	25.4 ± 3.2	24.7 ± 2.3	20.5 ± 4.3	0.392

*Data are expressed percentage as mean ± SD. The each patient number is presented in parenthesis. ^§^Derived from Chi-squared test*.

### Ultrasonography

Participants underwent measurement of carotid artery IMT, plaque morphology and thickness of carotid plaques via B-mode ultrasonography. Carotid artery plaques were examined using a high-resolution B-mode ultrasound system (Accuvix V10, Seoul, South Korea) equipped with a multi-frequency linear array transducer (5–10 MHz). All measurements were performed by a technician trained in ultrasound research in accordance with a standard scanning and reading protocol.

Carotid artery IMT is defined as a distance from media-adventitia to lumen-intima interface. Carotid artery IMT (longitudinal view) was measured offline in a plaque-free region at the far wall of the common carotid artery (CCA) using a computerized system. The upper limit of normal for IMT was defined as 1.0 mm. IMT measures were obtained from walls of three arterial segments of both carotid arteries; the near and far wall of the proximal 10 mm of the internal carotid artery, the near and far wall of the carotid bifurcation beginning at the tip of the flow divider and extending 10 mm proximally, and the near and far wall of the arterial segment extending 10–20 mm proximally to the tip the flow divider into the CCA (Prati et al., 2008).

In accordance with the Mannheim Consensus, carotid plaques were defined as focal protruding structures into the lumen with a size of at least 0.5 mm or 50% of the IMT where is over 1.5 mm (Touboul et al., 2007). The lesions with an IMT ≥ 1.1 mm were defined as atheromatous plaques. Thickness of carotid atheroma to measure and count the plaques was measured in a longitudinal and vertical view for screening accurate plaque. The plaques were measured everywhere.

### Flow Cytometry

Blood samples were collected into Vacutainer tubes containing 0.5 mL of 3.2% buffered sodium citrate, immediately following which the citrated blood sample was added to a fixation solution to minimize *ex vivo* platelet activation and prepared for flow cytometry. Whole blood samples were used to assess platelet activity. Whole blood resuspended in Tyrode’s solution was incubated with phycoerythrin (PE)-conjugated anti-CD41a for immunological identification of platelets. The samples were simultaneously incubated with fluorescein isothiocyanate (FITC)-conjugated anti-CD62p or FITC-conjugated anti-CD162 at saturating concentrations for 15 min at room temperature in the dark. Levels of platelet-bound anti-CD62p or -CD162 were determined by analyzing 50,000 platelets for FITC fluorescence. Results were expressed as a percentage of antibody-positive platelets.

Red blood cell-lysed blood samples were used to determine leukocyte levels, which were identified based on the forward and sideward scatter properties of PE-CD45-positive leukocytes. Monocytes and lymphocytes were identified based on strong expression of PE-CD14 and PE-CD154, respectively. The presence of platelet-leukocyte aggregates was assessed during the detection of CD42b-FITC-labeled platelets. FITC-conjugated immunoglobulin G (IgG) and PE-conjugated IgG antibody were used for isotype control experiments. A minimum of 50,000 cell events was analyzed in each assay. The percentages of platelet-monocyte and platelet-lymphocyte complexes were calculated. All antibodies used were purchased from BD Biosciences (New Jersey, NJ, USA). Blood samples were analyzed using LSRII (Becton Coulter, San Jose, CA, USA).

### Statistical Analysis

Participant characteristics and plaque thickness are presented as mean ± SD. Fluorescence-activated cell sorting (FACS) and ELISA values are presented as medians. The clinical characteristics of each group were compared using the Mann–Whitney nonparametric test. Statistical significance among groups was determined via one-way analysis of variance (ANOVA). Probability values were two-tailed, and values of *p* < 0.05 were considered statistically significant (SPSS, Windows version 17.0, Chicago, IL, USA). Pearson correlation coefficients were used to assess the association between ultrasound findings (IMT and plaque number) and changes in blood factors associated with atherosclerotic vessel dysfunction. Values of the coefficient constant *r* and *r*^2^ are provided. A value of *p* ≤ 0.05 was regarded as significant (SPSS, Windows version 17.0, Chicago, IL, USA).

## Results

### Clinical Characteristics and Ultrasound Findings of Control and Patient Groups

**Figure 1:**
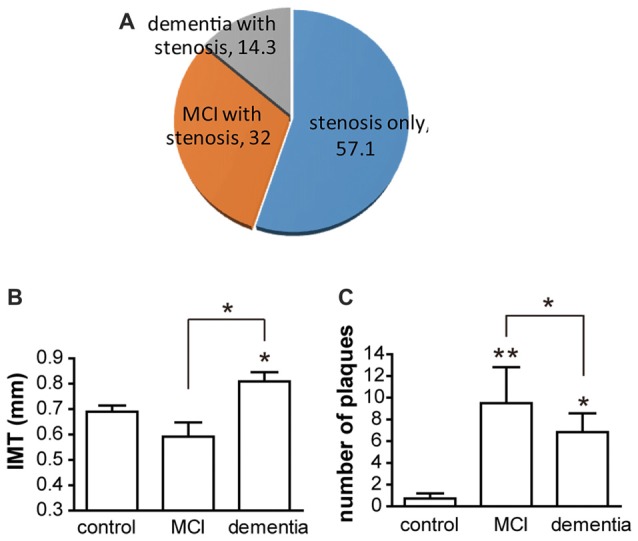
The distribution of participants and atherosclerotic condition. **(A)** Of participant, patients groups are three. Without cognitive decline, patients with atherosclerosis only are 57.1% of total patients. With atherosclerosis, patients with mild cognitive impairment (MCI) are composed in 32.1% and patients with dementia are constituted in 14.3%. **(B)** Intima-media thickness (IMT) is shown as mean ± SD. Differences of IMT is significant between dementia and control group. Also it is significant between dementia and MCI. **(C)** Numbers of plaques are shown as mean ± SD. There are significant differences between control and MCI; control and dementia; and MCI and dementia. *p < 0.05 vs. control group, **p < 0.01 vs. control group.

In the present study, there was no significant difference in vascular risk factors among the control, vessel damage, MCI and dementia groups. Moreover, there were no significant differences among the three groups with regard to total cholesterol (*p* = 0.4), HDL (*p* = 0.7421) and LDL (*p* = 0.3439). Participant characteristics are detailed in Table 1. Of all patients with vessel damage diagnosed with carotid vascular stenosis or atherosclerosis, patients with vessel damage without any cognitive dysfunction comprise 57.1%; patients with MCI is 32.1%; and patients with dementia is 14.3% (Figure 1A). Particularly, all of patient with MCI had been also with vessel damage. The IMT was significantly thicker in the dementia (mean thickness: 0.809 ± 0.097 mm) group than in the control group (mean thickness: 0.643 ± 0.123 mm), but not in the MCI group (mean thickness: 0.743 ± 0.226 mm; Figure 1B). However, plaque numbers were significantly increased in both the MCI and dementia groups relative to values for the control group (control: 2.5 ± 0.7 vs. MCI: 9.5 ± 6.6, *p* = 0.0061; control vs. dementia: 6.8 ± 4.2, *p* = 0.0012; Figure 1C).

### Changes in Levels of Circulating Adhesion Blood Factors in Patients with MCI and Dementia

**Figure 2:**
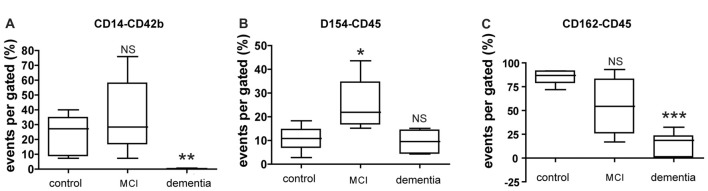
Expression of circulating blood factors by fluorescence-activated cell sorting (FACS) analysis. **(A)** Circulating levels of monocyte aggregated with platelets in each group. **(B)** Circulating levels of leukocyte aggregated with CD40Ligand (CD154) in each group. **(C)** Circulating levels of leukocyte aggregated with p-selectin glycoprotein ligand (PSGL; CD162) in each group. The data presents with % as an event number per gate by FACS analysis. Bars represent median values, and the error bar, the standard deviation. *p < 0.05 vs. control group, **p < 0.01 vs. control group, ***p < 0.001 vs. control group. NS, not significant.

FACS analysis of circulating blood factors associated with platelet-leukocyte aggregation (PLA) revealed that specific changes in PLA level in each MCI or dementia group (Figure 2). The levels of platelet-monocyte aggregates (PMA) as determined by CD154-CD14 double-staining is lower in dementia group compared to control, but not significant difference in MCI group (Figure 2A). In contrast, the level of PLA (CD154-CD45) is higher in MCI group, but not significant change in dementia group (Figure 2B). In case of another PLA (CD162-CD45), there was significant decrease in dementia group compared to control. It was different from the result in MCI group presenting no significant change compared to control (Figure 2C).

**Figure 3:**
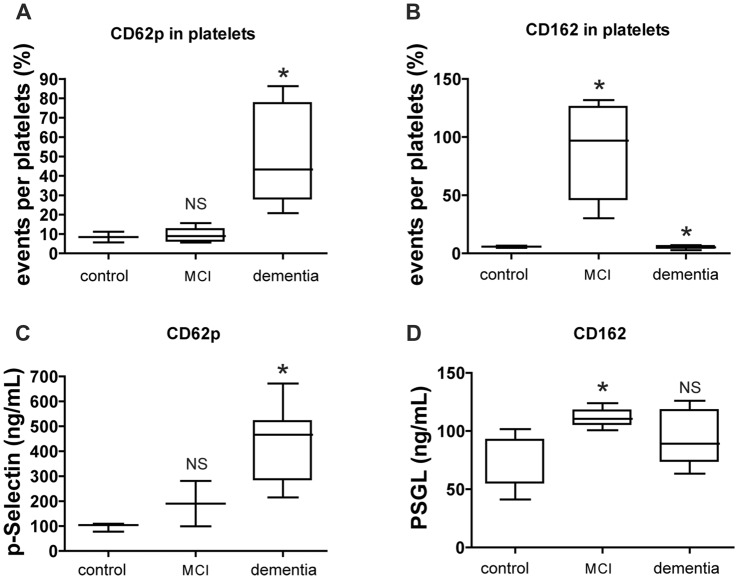
CD62p and CD162 expression in cell surface marker and soluble level. **(A,B)** Level of platelet surface expressing CD62p (p-selectin) or CD162 (PSGL) was evaluated by FACS analysis. **(C,D)** Level of CD62p or CD162 in plasma was evaluated by ELISA. The data of FACS represents with % as an event number per gate. The result of ELISA is presented as ng/ml. Bars represent median values, and the error bar, the standard deviation. *p < 0.05 vs. control group. NS, not significant.

The number of platelets expressing p-selectin (CD62p) by FACS was increased in dementia group whereas the number of platelets expressing p-selectin glycoprotein ligand (PSGL; CD162) was high both in MCI and dementia group (Figures 3A,B). The level of p-selectin detected on the surface of active platelets significantly increased in the vessel damage group and MCI group, based on changes in platelet-rich plasma (PRP) levels as determined via ELISA. The level of p-selectin and PSGL in plasma was higher in the MCI group than in the control group (Figures 3C,D).

### Associations between Vessel Status and Circulating Blood Factors in Patients with Cognitive Decline

The correlation between cognitive function and circulating adhesion factors was analyzed as shown in Table 2. The number of platelets expressing p-selectin was correlated with IMT and plaque numbers in both the MCI and dementia groups. The number of platelets expressing PSGL was strongly correlated with IMT, but not with plaque numbers, in MCI group. In contrast, the number of platelets expressing PSGL was correlated with plaque number rather than IMT in dementia group. Unlike in the dementia group, factors associated with blood aggregation and vessel dysfunction such as CD40Ligand (CD154), PLA and PMA, were correlated with IMT and plaque number in the MCI group (Table 2). In the MCI group, IMT was significant correlation with most of circulation blood factors except of the level of PMA. Plaque number in MCI group was also significantly correlated with p-selectin, PLA and PMA (Table 2). In the dementia group, IMT was significant correlation with the level of p-selectin only. In contrast, plaque number was a significant correlation with p-selectin and strong correlation with PSGL among circulating adhesion factors (Table 2).

**Table 2:** Correlation between ultrasonic characters and circulating blood factors.

Correlating pair		MCI			Dementia		
Ultrasonic characters	Circulating blood factors	*r*	*R*^2^	*p* value	*r*	*R*^2^	*p* value
IMT	p-selectin	0.810	0.656	0.0149*	0.609	0.371	0.0273*	
	PSGL	0.951	0.904	0.0036**	−0.207	0.043	0.5653	
	CD40 Ligand	0.906	0.822	0.0019**	−0.035	0.001	0.923	
	PLA	0.819	0.671	0.0243*	0.333	0.111	0.2667	
	PMA	0.370	0.137	0.3665	0.191	0.037	0.5317	
Number of plaques	p-selectin	0.954	0.911	0.0116*	0.665	0.442	0.0255*	
	PSGL	0.775	0.600	0.0704	0.921	0.849	0.0002***	
	CD40 Ligand	0.333	0.111	0.4656	0.565	0.319	0.0887	
	PLA	0.808	0.653	0.0278*	−0.026	0.001	0.9434	
	PMA	0.905	0.819	0.0347*	−0.388	0.150	0.1906	

Pearson’s correlation coefficients, R squared, and associated p-values are shown. The bold values are representing the significant circulating blood factors in each ultrasonic character group. *p < 0.05 vs. control group, **p < 0.01 vs. control group, ***p < 0.001 vs. control group.

## Discussion

In the present study, we demonstrated that alterations in IMT and plaque number are associated with an increased risk of cognitive decline as well as risk of dementia. Our results suggest that ultrasound findings may aid in identifying older individuals at increased risk for the progression of cognitive decline when morphological impairment of cerebrovascular structures has been identified. Moreover, our findings suggest that the presence of atherosclerotic changes and changes in blood factors such as PSGL, PLA and PMA can be used to predict MCI and dementia.

In the present study, levels of p-selectin in circulating platelets, PSGL and circulating PMA were significantly increased in patients with MCI relative to controls (Figures 2, 3). The changes in circulating blood factors have been reported to relate with vascular diseases like as ischemic stroke or atherosclerosis (Marquardt et al., 2002; Nadar et al., 2004). Based on this association, several noninvasive measures for evaluating subclinical atherosclerosis have received intense attention in clinical and research settings for the predictive diagnosis of cerebrovascular diseases.

Researchers have suggested a relationship between atherosclerotic severity and circulating adhesion blood factors; atherosclerotic severity and cognitive decline in the above mentioned reports. With one step further linking between them, our findings provide insight into the use of blood factor analysis (using FACS) as well as ultrasonographic evaluation of vessel status in both clinical and research settings. Changes in platelet activation and monocyte distribution are observed in the early stages of atherosclerosis. Such changes are strongly associated with stroke onset, as demonstrated by various studies (King et al., 2009; Cardenas et al., 2012; Xiang et al., 2013). The monocyte receptor CD14 and leukocyte antigen CD45 are best known for their crucial role in immunity. In addition, CD14 and CD16 are well-known biomarkers for atherosclerotic disease progression (Folsom et al., 2009). Research has also suggested that (PSGL, CD162) is a pro-atherogenic marker of vascular disease progression (Folsom et al., 2009).

The present study shows that increased IMT was more frequently observed in patients with MCI, whereas increased numbers of carotid plaques were more frequently observed in patients with dementia. The patients with MCI in our study comprise 32% of all patients with atherosclerosis, and all patients of the MCI group in the present study had been diagnosed with carotid vascular stenosis or atherosclerosis (Figure 1). These findings suppose that vessel damage is followed by MCI. A lot of findings in previous studies suggest that greater degrees of carotid atherosclerosis are associated with the progression from MCI to dementia (Mioshi et al., 2006; Urbanova et al., 2014; Moon et al., 2015; Knopman et al., 2016; Dearborn et al., 2017). The very recent study reported that up to 50% of patients develop vascular stenosis, and that anterior cerebral artery (ACA) plaques are associated with dementia even after controlling for vascular risk factors (Dearborn et al., 2017). Other researches have suggested that atherosclerosis plays a role in cognitive impairment, particularly in older adults (Jellinger and Attems, 2007; White et al., 2016). Such research has further demonstrated a converging relationship between degenerative vascular dysfunction and cognitive dysfunction. In our study, most patients with MCI exhibit atherosclerotic vessel abnormalities, such as increased IMT and plaque numbers, increasing the risk for progression to dementia. Especially in aged people, it is reported to estimate that 15%–42% of people over the age of 65 years exhibit some form of MCI, and that approximately 5%–15% of patients with MCI go on to develop dementia (Winblad et al., 2004; Petersen et al., 2014). Recent evidence has revealed that vessel dysfunction contributes to AD as well as vascular dementia (Murray et al., 2011). In this previous study, the authors reported an IMT cutoff value of 0.805 for the prediction of MCI development (baseline: 0.825 mm; Murray et al., 2011). Diagnosis of dementia in such patients is required in order to ensure the appropriate therapeutic guidelines and treatment are utilized (Wu et al., 2017).

Our results indicate that intima thickness and plaque number are associated with higher levels of p-selectin, as the evidence that platelets are engaged in the formation of PLAs (Steinhubl et al., 2007). In the dementia group of the present study, which included individuals with dementia, plaque numbers corresponded strongly with levels of PSGL-positive platelets. Control of plaque numbers with appropriate therapy such as statin treatment may thus delay or prevent the progression of cognitive decline to dementia. Our findings also indicated that carotid atherosclerosis correlates with MCI as well as increased numbers of PSGL-expressing platelets. Analysis of blood factors using ultrasonography may aid clinicians in determining the most appropriate treatment strategy for patients with cognitive decline with vessel disease.

The present study has some limitations that need consideration. First, longer follow-up is required to verify our findings. We are currently preparing a 3-year follow-up study including the participants of the present study and additional volunteers. Second, as the sample size was rather small, future studies should enroll a larger population. Future FACS studies should also aim to determine a cutoff value for each blood cell population for the prediction of progression from cognitive decline to dementia in older adults with cardio/cerebrovascular disease. Our simple assessment of vascular risk factors does not seem to be a fully satisfactory approach for adequately counteracting the risk of developing dementia, when compared to other large-scale studies (Coley et al., 2008; Luzzi et al., 2010). Nevertheless, we suggest that analysis of circulating adhesion factors may aid in predicting the risk of progressive cognitive impairment. Additionally, aggressive treatments for vascular disease should be considered for individuals with a predisposition toward dementia. Despite these limitations, our findings provide a basis for further study regarding biomarkers of both cerebrovascular disease and cognitive dysfunction.

In conclusion, our findings demonstrate that circulating adhesion molecules level and interaction between factors present significant differences in patient with MCI or dementia. Alterations in IMT and plaque number are associated with an increased risk of cognitive decline as well as conversion from MCI to dementia. These results suggest that ultrasound findings may aid in identifying older individuals at increased risk for the progression of cognitive decline in when cerebrovascular damaged. Moreover, our findings suggest that the presence of atherosclerotic changes and changes in blood factors such as p-selectin, PSGL, PLA and PMA can be useful candidates to monitor the severity of cognitive decline.

## Author Contributions

KC composed the manuscript and performed most of laboratory works and data. JK collected blood sample from outpatients and clinical records; and GWK supervised the whole study.

## Conflict of Interest Statement

The authors declare that the research was conducted in the absence of any commercial or financial relationships that could be construed as a potential conflict of interest.

## Abbreviations

AD: Alzheimer’s disease
ANOVA: analysis of variance
CDR: Clinical Dementia Rating Scale
FACS: fluorescence-activated cell sorting
FITC: fluorescein isothiocyanate
HDL: high-density lipoprotein
IgG: immunoglobulin G
IMT: intima-media thickness
LDL: low-density lipoprotein
MCI: mild cognitive impairment
MMSE: Mini-Mental State Examination
PLA: platelet-leukocyte aggregation
PMA: platelet-monocyte aggregation
PRP: platelet-rich plasma
PSGL: p-selectin glycoprotein ligand.
