# Corrigendum: Changes in Blood Factors and Ultrasound Findings in Mild Cognitive Impairment and Dementia

**DOI:** 10.3389/fnagi.2018.00033

**Published:** 2018-02-13

**Authors:** Kyoungjoo Cho, Jihye Kim, Gyung W. Kim

**Affiliations:** Department of Neurology, College of Medicine, Yonsei University, Seoul, South Korea

**Keywords:** dementia, vascular disease, mild cognitive impairment, blood factor analysis, atherosclerosis

In the published article, the first affiliation was incorrect. Instead of “(Department of Life Science, Kyonggi University, Suwon, South Korea)”, it should be “(Department of Neurology, College of Medicine, Yonsei University, Seoul, South Korea)”. The authors apologize for this error and state that this does not change the scientific conclusions of the article in any way.

The incorrect affiliation has been removed and the article has been updated.

## Conflict of interest statement

The authors declare that the research was conducted in the absence of any commercial or financial relationships that could be construed as a potential conflict of interest.

